# Effect of Seed Position on Parental Plant on Proportion of Seeds Produced with Nondeep and Intermediate Physiological Dormancy

**DOI:** 10.3389/fpls.2017.00147

**Published:** 2017-02-09

**Authors:** Juan J. Lu, Dun Y. Tan, Carol C. Baskin, Jerry M. Baskin

**Affiliations:** ^1^College of Grassland and Environment Sciences, Xinjiang Agricultural UniversityUrümqi, China; ^2^Department of Biology, University of Kentucky, LexingtonKY, USA; ^3^Department of Plant and Soil Sciences, University of Kentucky, LexingtonKY, USA

**Keywords:** Brassicaceae, cold stratification, *Isatis violascens*, seed dormancy, silicles

## Abstract

The position in which seeds develop on the parental plant can have an effect on dormancy-break and germination. We tested the hypothesis that the proportion of seeds with intermediate physiological dormancy (PD) produced in the proximal position on a raceme of *Isatis violascens* plants is higher than that produced in the distal position, and further that this difference is related to temperature during seed development. Plants were watered at 3-day intervals, and silicles and seeds from the proximal (early) and distal (late) positions of racemes on the same plants were collected separately and tested for germination. After 0 and 6 months dry storage at room temperature (afterripening), silicles and seeds were cold stratified for 0–16 weeks and tested for germination. Mean daily maximum and minimum temperatures during development/maturation of the two groups of seeds did not differ. A higher proportion of seeds with the intermediate level than with the nondeep level of PD was produced by silicles in the proximal position than by those in the distal position, while the proportion of seeds with nondeep PD was higher in the distal than in the proximal position of the raceme. The differences were not due only to seed mass. Since temperature and soil moisture conditions were the same during development of the seeds in the raceme, differences in proportion of seeds with intermediate and nondeep PD are attributed to position on parental plant. The ecological consequence of this phenomenon is that it ensures diversity in dormancy-breaking and germination characteristics within a seed cohort, a probable bet-hedging strategy. This is the first demonstration of position effects on level of PD in the offspring.

## Introduction

According to the Nikolaeva-Baskin classification system, there are five classes of seed dormancy, i.e., physiological dormancy (PD), morphological dormancy (MD), morphophysiological dormancy (MPD), physical dormancy (PY), and combinational dormancy (PY + PD) (Baskin and Baskin, 2014). PD is caused by low growth potential of the embryo, and it occurs in three increasing degrees or depths (intensities) of dormancy as follows: nondeep PD < intermediate PD < deep PD. Nondeep PD can be broken by high (≥15°C) or low (0–10°C and wet, i.e., cold stratification) temperatures, depending on the species, and it is the most common kind of seed dormancy on Earth. In temperate regions of the world, intermediate and deep PD can be broken by long periods of cold stratification. However, exposure of seeds with intermediate PD to high temperatures for 2–3 months before the beginning of cold stratification significantly decreases the length of the cold treatment required to break dormancy (Baskin and Baskin, 2014).

Seeds produced under different temperature conditions can vary in intensity of PD. Generally, seeds produced at high temperatures are less dormant than those produced at low temperatures (Baskin and Baskin, 2014), but there are a few exceptions for which the reverse is true (Koller, 1962; Groves et al., 1982). Seeds of some species, including those of *Thlaspi arvense* (Brassicaceae) (Hume, 1994), collected in the early part of the growing season when temperatures are relatively low, are more dormant than those collected late in the growing season when temperature are relatively high (Baskin and Baskin, 2014, p. 326). However, germination percentage of early- and late-collected seeds of *Brassica campestris* was the same (Singh et al., 1976).

In a seed cohort of *Isatis violascens* Bunge (Brassicaceae), there are two levels of PD: nondeep and intermediate (Zhou et al., 2015). Seeds with nondeep PD afterripen (come out of dormancy) during summer and germinate in autumn if the soil is moist; otherwise, germination is delayed until next spring. Seeds with intermediate PD require ≥ 12 weeks of cold stratification for dormancy break; however, a period of dry storage (afterripening) significantly decreases the length of the cold stratification period required to break dormancy. In the field, seeds with intermediate PD afterripen in summer, are cold stratified in winter and germinate in spring. Seeds with nondeep PD can germinate in autumn or spring, while those with intermediate PD can germinate only in spring (Lu et al., 2016). In a seed cohort of *I. violascens* collected in 2013, 20–25% of the seeds had nondeep PD and 75–80% intermediate PD (Zhou et al., 2015).

Timing and position of seed development on the parental plant can affect the intensity of PD (Hume, 1994; Baskin and Baskin, 2014). Thus, we hypothesized that plants of *I. violascens* produce seeds with intermediate PD under relatively cool conditions in early spring (proximal position on raceme) and seeds with nondeep PD under relatively warm conditions in mid to late spring (distal position on raceme). To address this hypothesis, we asked: does the proportion of nondeep and intermediate PD differ, depending on time, position and/or temperature during seed development/maturation.

*Isatis violascens* is an annual, native to central Asia, and in China it grows on sand dunes in the Garbantunggut Desert of Xinjiang (Zhou et al., 2011). Indehiscent intact silicles are the dispersal and germination units of this species (Liu and Tan, 2007). Each silicle has a membranous wing around it and contains a single seed. Each plant produces one to five racemes with a total of 15–200 silicles, depending on size of the plant (Zhou et al., 2015). Thus, early produced silicles/seeds were collected from a large number of plants, and late-produced silicles/seeds subsequently were collected from the same plants.

## Materials and Methods

### Silicle Production and Description

In early June 2013, dispersal units (i.e., silicles) were collected from about 1000 plants growing in a natural population of *I. violascens* on a cold desert sand dune in Fukang City in the southern part of the Junggar Basin of Xinjiang Province (44°22′ N, 88°08′ E, 458 m a.s.l.), China. Approximately 10,000 silicles were sown in an experimental garden on the campus of Xinjiang Agricultural University in Urümqi, on the southern edge of the Junggar Basin (43°53′ N, 87°33′ E, 696 m a.s.l.). This population has regenerated each year after sowing, and in 2015, when silicles were collected for this study, it contained about 1000 plants.

**FIGURE 1:**
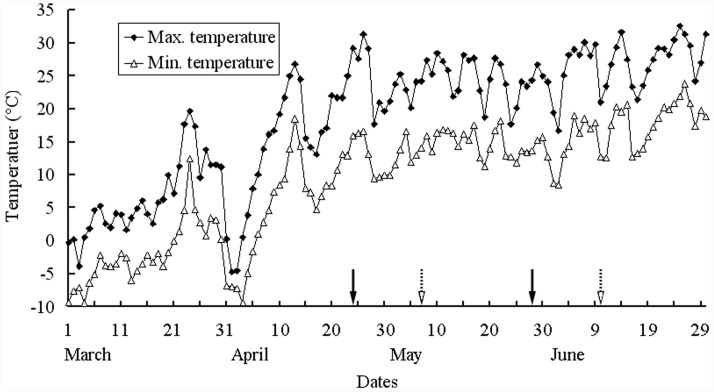
**Mean maximum and minimum temperatures from March to June 2015 in Urümqi, China.** Arrows with solid and broken lines are the periods from flowering to silicle maturation for proximal and distal positions on racemes of *Isatis violascens*, respectively. The weather data were provided by Geographical Information Monitoring Cloud Platform.

During the 2015 germination and plant growth season (i.e., 18 March to 10 June for *I. violascens*), the soil was watered to field capacity every 3 days to ensure that water was not a limiting factor for seed germination, seedling survival, pre-reproductive growth and seed production and development. During seed development, approximately 800 plants were selected for collection of mature silicles. The first green silicle from the proximal position on 2–5 racemes on each of the 800 plants was marked with red nail polish on 1 May 2015. Fourteen days later (15 May 2015), the first green silicle from the distal position on the same racemes on the same plants was marked with blue nail polish. Each of the two groups of silicles was marked 6–7 days after pollination. Before dispersal, we collected the mature silicles from the proximal (early maturation) and the distal (late-maturation) positions on racemes on 28 May (i.e., 34 days after pollination) and 10 June 2015 (i.e., 34 days after pollination), respectively. The two groups of silicles were stored in separate paper bags at room conditions (16–30°C, 10–40% RH) until used. Mean maximum and minimum air temperatures from seed germination to fruit maturity (i.e., March to June 2015) are shown in **Figure 1** (National Meteorological Information Center, China Meteorological Administration, http://cdc.nmic.cn/).

### Mass of Silicle, Seed, Pericarp, Embryo, and Seed Coat

Five replications of five intact silicles, of five seeds, of pericarps from five silicles, of five embryos and of seed coats from five seeds from distal and proximal positions on racemes were weighed individually using a Sartorius BS210S electronic-balance (0.0001 g).

### Presence of Nondeep PD

By conducting tests for nondeep and intermediate PD in seeds from both distal and proximal positions on the racemes, we can determine if the proportion of nondeep and intermediate PD differs for seeds matured at different times and positions. If seeds of *I. violascens* have nondeep PD, they will afterripen during dry storage at room temperature (Zhou et al., 2015). After silicles from distal and proximal positions on racemes had been stored dry in the laboratory for 0 (i.e., June 2015) and 6 months (i.e., December 2015), intact silicles and isolated seeds from distal and proximal positions on racemes were incubated in Petri dishes on wet filter paper at 5/2°C in darkness, the optimum conditions for germination (Zhou et al., 2015). Four replicates of 25 intact silicles and of 25 isolated seeds each from distal and proximal positions on racemes were used to test germination. Silicles and seeds were checked only after 28 days; therefore, they were not exposed to any light during the incubation period.

After the germination trials were complete, the non-germinated seeds were tested for viability. Seeds were cut open and the embryo observed. Seeds with white, firm embryos were counted as viable, and those with tan, soft embryos were considered nonviable and excluded from the calculations of germination percentages. Only a very few seeds were non-viable. The tests of fresh seeds (0 month old) from distal and proximal positions on racemes were initiated on 31 May and 12 June 2015, respectively, using seeds collected on 28 May and 10 June 2015, respectively.

### Presence of Intermediate PD

Seeds of *I. violascens* with intermediate PD will germinate after they have been dry stored (afterripened) for 6 months and then given a cold stratification treatment (Zhou et al., 2015). To determine the proportion of seeds from distal and proximal positions on racemes that had intermediate PD, we used seeds and silicles from these two positions that had been stored dry at room temperature for 6 months. Six-month-old intact silicles and isolated seeds from distal and proximal positions on racemes were cold stratified on moist filter paper at 4°C for 0, 4, 8, 12, and 16 weeks. After each period of cold stratification, four replications of 25 silicles and of 25 seeds from both positions were checked for germination. Then, four replicates of 25 intact silicles and of 25 isolated seeds from both positions that did not germinate after each period of cold stratification were tested for germination at 5/2°C in darkness for 28 days. Since >90% of intact silicles and of isolated seeds germinated during the 16 weeks of cold stratification, no germination test *per se* was performed.

### Statistical Analysis

Independent-sample *t*-test were used to compare the differences in silicle, seed, pericarp, embryo, and seed coat mass between distal and proximal positions on racemes. Germination data were analyzed using generalized linear models (GLMs) with a logit link to germination as a binomial response variable (two categories: germinated versus non-germinated). In the models, silicle position (distal and proximal positions on racemes), treatment (intact silicles and isolated seeds) and storage time (0 and 6 months) were used as fixed factors for the “Presence of nondeep PD” experiment, and silicle positions (distal and proximal positions on racemes), treatment and cold stratification time (0, 4, 8, and 12 weeks) were used as fixed factors for the “Presence of intermediate PD” experiment, with their interactions included in the models. The significance of effects of fixed factors and their interactions in the models was tested by Wald χ^2^ values. Tukey’s HSD test was performed for multiple comparisons to determine significant differences among treatments. Correlative analyses were used to determine the relationship between embryo mass and germination percentage of 6-month-old dry-stored seeds from distal and proximal positions on racemes. With mass of seeds as a covariate, a one-way ANCOVA was used to determine whether the proportion of seeds with intermediate and nondeep PD differed between seeds produced in the distal and proximal position of the raceme. Statistical tests were conducted at *P* = 0.05. All data analyses were performed with the software SPSS 16.0 (SPSS, Inc., Chicago, IL, USA). Values are mean ± 1 SE (i.e., standard errors).

## Results

### Mass of Silicle, Seed, Pericarp, Embryo, and Seed Coat

Mass of whole silicles, whole seeds, and embryos from the proximal position on the raceme was significantly greater than that of silicles, seeds, and embryos from the distal position However, there was no significant difference in mass of pericarps or seed coats between these two positions on raceme (**Table 1**).

**Table 1:** Effects of silicle position on silicle, seed, pericarp, embryo, and seed coat mass in *Isatis violascens* (mg, mean of five individuals ± 1 SE).

	DR	PR
Silicle	37.66 ± 0.59^a^	42.00 ± 0.34^b^
Seed	18.90 ± 0.36^a^	22.82 ± 0.15^b^
Pericarp	18.76 ± 0.23^a^	19.18 ± 0.38^a^
Embryo	17.9 ± 0.10^a^	21.86 ± 0.43^b^
Seed coat	1.76 ± 0.12^a^	2.32 ± 0.29^a^

*DR, distal position on raceme; PR, proximal position on raceme. Different lowercase letters in a row indicate significant differences (P < 0.05) between DR and PR by t-test.*

### Presence of Nondeep PD

**FIGURE 2:**
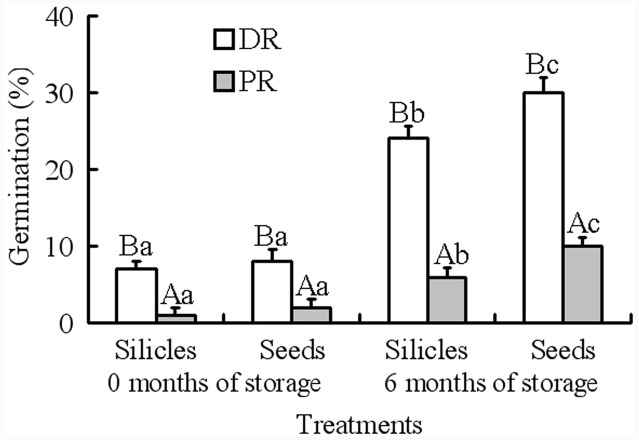
**Germination of 0- and 6-month-old silicles and seeds from distal and proximal positions on racemes (mean + 1 SE) of *Isatis violascens* incubated in darkness at 5/2°C.** Different lowercase letters indicate significant differences (*P* < 0.05) after 0 and 6 months of storage and for different pericarp treatments (intact fruit versus isolated seed) for distal and proximal positions on racemes and different uppercase letters significant differences between distal and proximal positions on raceme at the same storage time and for the same pericarp treatment. DR, distal position on raceme; PR, proximal position on raceme.

Generalized linear models analysis showed that germination was significantly affected by silicle position on raceme and storage time; however, the effect of treatment (i.e., intact silicles and isolated seeds) and of the various interactions was not significant (**Table 2**). At storage time zero, germination of seeds in intact silicles and isolated seeds from distal and proximal positions on the racemes was 1–8% (**Figure 2**). After 6 months of dry storage, 24 and 30% of seeds in silicles and isolated seeds from the distal position on the racemes germinated, respectively, but only 6 and 10% of those from the proximal position on the racemes germinated, respectively (**Figure 2**).

**Table 2:** Generalized linear models of effects of silicle position on raceme (P), treatment (T), storage time (S), and their interactions on presence of nondeep PD and of silicle position on raceme (P), treatment (T), cold stratification time (C), and their interactions on presence of intermediate PD in *Isatis violascens*.

Factor	d.f.	Wald-χ^2^	*P*-value
**Presence of nondeep PD**		
P	1	18.652	<0.05
T	1	1.325	0.250
S	1	19.598	<0.05
P × T	1	0.297	0.586
P × S	1	0.118	0.731
T × S	1	0.000	0.994
P × T × S	1	0.044	0.834
**Presence of intermediate PD**		
P	1	167.519	<0.05
T	1	5.508	<0.05
C	3	178.883	<0.05
P × T	1	0.027	0.868
P × C	3	1.643	0.650
T × C	3	0.424	0.935
P × T × C	3	0.232	0.972

### Presence of Intermediate PD

**FIGURE 3:**
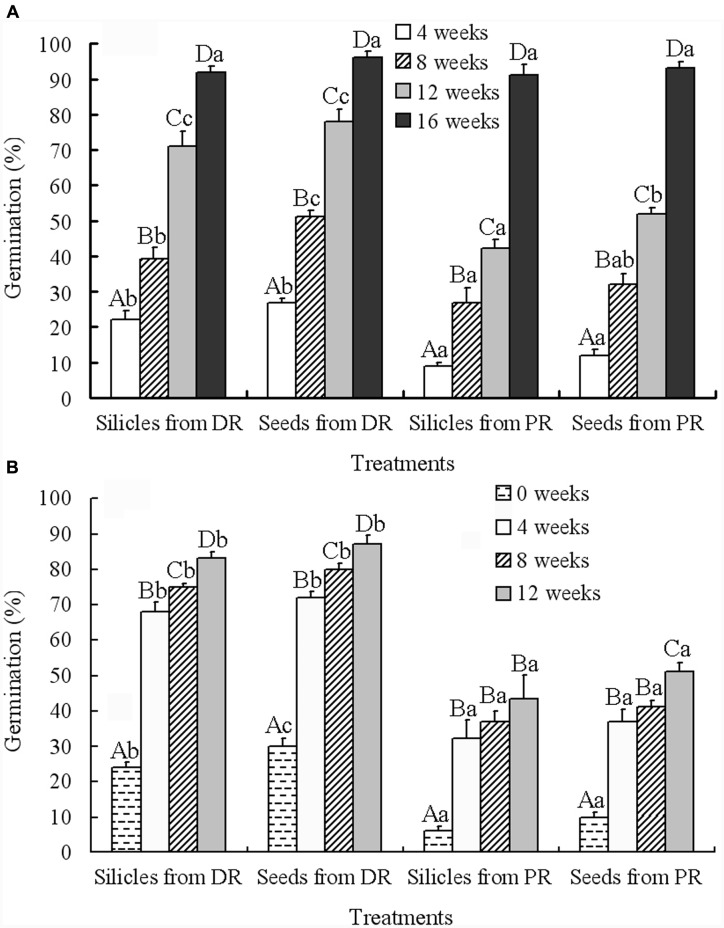
**Effect of cold stratification on germination (mean + 1 SE) of 6-month-old dry-stored silicles and seeds from distal and proximal positions on racemes of *Isatis violascens* during cold stratification at 4°C for 4, 8, 12, and 16 weeks (A)** and during incubation at 5/2°C in darkness after cold stratification for 0, 4, 8, and 12 weeks **(B)**. Different uppercase letters indicate significant differences (*P* < 0.05) among different treatments at the same cold stratification time and different lowercase letters significant differences among different cold stratification times within the same treatment. DR, distal position on raceme; PR, proximal position on raceme.

Generalized linear models analysis showed that the effects of silicle position on raceme, treatment (i.e., intact silicles and isolated seeds) and cold stratification time on germination were highly significant, but none of the interactions had significant effects (**Table 2**). With increase in cold stratification time, germination of seeds in silicles and isolated seeds from distal and proximal positions on the racemes increased significantly, and they germinated to 93–96% during 16 weeks at 4°C (**Figure 3A**). After 12 weeks of cold stratification at 4°C, at time of transfer to 5/2°C, germination of seeds in silicles and isolated seeds from the distal position of the racemes was 83 and 87%, respectively, and that of seeds in silicles and isolated seeds from the proximal position of racemes was 43 and 51%, respectively (**Figure 3B**). However, after 16 weeks of cold stratification at 4°C, >90% of the seeds from both positions had germinated (**Figure 3A**); thus, a germination test was not performed.

Additionally, the results of correlative analyses showed that there was a significantly negative relationship between embryo mass and germination percentage of 6-month-old dry-stored seeds from distal positions on racemes (*r* = -0.948, *P* < 0.001), but a positive relationship between embryo mass and germination percentage of 6-month-old dry-stored seeds from proximal positions (*r* = 0.948, *P* < 0.001). Also, a one-way ANCOVA indicated a significant effect of seed position on the raceme on proportion of seeds with intermediate and nondeep PD (*P* < 0.05).

## Discussion

Our hypothesis that plants of *I. violascens* produce seeds with intermediate PD in early spring and seeds with nondeep PD in mid- to late spring was supported in part. That is, while the proportion of seeds with intermediate PD was higher in the proximal (early) part of the raceme (90%) than in the distal (late) part (70%), some seeds with nondeep PD (10%) were produced in the proximal position. Also, while the proportion of seeds with nondeep PD was higher in the distal (30%) than proximal (10%) position of the raceme, seeds with intermediate PD (70%) also were produced in the distal position (**Figures 2** and **3B**). Thus, seeds with nondeep and intermediate PD are produced in both the proximal and distal positions of the raceme, but the proportion of seeds with the two levels of PD varies between the two positions.

During the 34-day period from flowering to maturation of seeds in both the distal and proximal positions of the raceme, plants were well watered, and mean daily maximum and minimum temperatures were 24.4/14.1 and 24.9/14.7°C, respectively, for the two periods of development. Thus, discounting the relatively small differences in day length and photon irradiance during the two periods of maturation, growth conditions of the plants while seeds developed in the two positions on the racemes were the same. It should be noted that 22 days of the two periods of maturation overlapped with each other (**Figure 1**). Thus, the major difference between seeds produced in the proximal and distal positions on the raceme was position *per se* on the mother plant.

Various studies have been conducted on the effects of the position of diaspore monomorphic fruit/seeds on the mother plant on seed mass and germination (Baskin and Baskin, 2014). In considering seeds produced in different parts of the same inflorescence, dormancy of seeds produced in the proximal part of the inflorenscence generally is greater than that of seeds produced in the distal part (e.g., Espadaler and Gomez, 2001; Wang et al., 2010). Thus, the higher proportion of intermediate than of nondeep PD in the proximal part of the raceme of *I. violascens* is consistent with results reported in the literature. In *Oldenlandia corymbosa*, however, seeds produced along the main axis of the inflorescence and at proximal ends of inflorescence branches were more dormant than these produced at distal ends of inflorescence branches (Do Cao et al., 1978).

Another consequence of position in the inflorescence is that proximal seeds often have greater mass than distal ones, e.g., the Brassicaceae species *Alliaria petiolata* (Susko and Lovett-Doust, 2000a,b) and *Brassica napus* (Clarke, 1979). However, seed mass was greater in distal than in proximal parts of the inflorescence of *Clarkia unguiculata* (Mazer and Dawson, 2001) and did not differ with inflorescence position in *Phytolacca rivinoides* (Byrne and Mazer, 1990). Since energy resources of the mother plant are limited, it is logical that seeds produced early in the season would have greater mass than those produced late in the season. Indeed, studies on a variety of plant taxa (see Baskin and Baskin, 2014, p. 355), including *Alliaria petiolata* (Susko and Lovett-Doust, 2000a), have found that seeds from early produced fruits have greater mass than those from late-produced fruits.

The greater dormancy and greater mass of seeds and embryo from the early, proximal position on the *I. violascens* raceme than from the later, distal position in general are consistent with results reported in the literature for various species, including some other Brassicaceae (Clarke, 1979; Susko and Lovett-Doust, 2000a,b). Even when mass of seeds was considered as a covariant in a one-way ANOVA, there was a significant position effect for a higher proportion of intermediate than nondeep PD for seeds produced in the proximal position of the raceme. This means that the higher proportion of intermediate than nondeep PD on the proximal position of the raceme was not due only to seed mass but to some other factor(s) associated with seed production in this position. What is new about our research is that for the first time we document that two levels of PD in seeds are produced in different positions on the same plants. Specifically, we show that seeds produced in the proximal position of the inflorescence are more likely to have intermediate PD and less likely to have nondeep PD than those on the distal position.

Seeds with intermediate PD produced in the proximal and distal positions on the racemes differed in the amount of cold stratification at 4°C required for dormancy break and germination following afterripening for 6 months. Whereas seeds from the distal position on the raceme germinated to 87% at 5/2°C after 12 weeks cold stratification at 4°C, those from the proximal positions germinated to only 51% (**Figure 3B**). However, seeds from the proximal position on the raceme had germinated to 96% after 16 weeks of incubation at 4°C (**Figure 3B**). Thus, 16 weeks of incubation at 4°C was more favorable for dormancy break than 12 weeks cold stratification at 4°C plus 4 weeks incubation at 5/2°C. The implication of the differences in intermediate PD between the proximally- and distally- produced seeds is that overall those produced in the proximal position had deeper intermediate PD than those produced late in the season.

## Conclusion

The germination/fitness strategy of *I. violascens* would appear to consist of both a conservative and a diversified bet-hedging strategy (Philippi, 1993): conservative because germination in both autumn and next spring (Zhou et al., 2015) avoids the risk of a complete failure of an autumn-germinating cohort of an annual species that has little potential to form a soil seed bank (Zhou et al., 2015); and diversified because a single plant produces seeds with two levels of PD that can germinate in two different seasons. The production of two levels of PD by plants of *I. violascens* may be one way in which a species that does not produce heteromorphic diaspores that differ in ecology and does not form a persistent seed bank can have a bet-hedging strategy.

## Author Contributions

JL and DT conceived and designed the experiments. JL performed the experiments and analyzed the data. JL, DT, CB, and JB wrote the manuscript. All authors reviewed and approved the manuscript.

## Conflict of Interest Statement

The authors declare that the research was conducted in the absence of any commercial or financial relationships that could be construed as a potential conflict of interest. The reviewer MF-V and handling Editor declared their shared affiliation, and the handling Editor states that the process nevertheless met the standards of a fair and objective review.
